# The cost-effectiveness of an outpatient anesthesia consultation clinic before surgery: a matched Hong Kong cohort study

**DOI:** 10.1186/2047-0525-1-3

**Published:** 2012-06-27

**Authors:** Anna Lee, Po Tong Chui, Chun Hung Chiu, Tony Gin, Anthony MH Ho

**Affiliations:** 1 Department of Anesthesia and Intensive Care, Prince of Wales Hospital, The Chinese University of Hong Kong, Shatin, New Territories, Hong Kong

**Keywords:** Cost-effectiveness analysis, Outpatient anesthesia clinic, Perioperative system, Patient satisfaction

## Abstract

**Background:**

Outpatient anesthesia clinics are well established in North America, Europe and Australia, but few economic evaluations have been published. The Perioperative Systems in Hong Kong are best described as a hybrid model of the new and old systems of surgical care. In this matched cohort study, we compared the costs and effects of an outpatient anesthesia clinic (OPAC) with the conventional system of admitting patients to the ward a day before surgery for their pre-anesthesia consultation. A second objective of the study was to determine the patient’s median Willingness To Pay (WTP) value for an OPAC.

**Methods:**

A total of 352 patients were matched (1:1) on their elective surgical procedure to either the clinic group or to the conventional group. The primary outcome was quality of recovery score and overall perioperative treatment cost (US$). To detect a difference in the joint cost-effect relationship between groups, a cost-effectiveness acceptability curve (CEAC) was drawn. A modified Poisson regression model was used to examine the factors associated with patients willing to pay more than the median WTP value for an OPAC.

**Results:**

The quality of recovery scores on the first day after surgery between the clinic and conventional groups were similar (mean difference, -0.1; 95% confidence interval (CI), -0.6 to 0.3; *P* = 0.57). Although the preoperative costs were less in the clinic group (mean difference, -$463, 95% CI, -$648 to -$278 per patient; *P* <0.001), the total perioperative cost was similar between groups (mean difference, -$172; 95% CI, -$684 to $340 per patient; *P* = 0.51). The CEAC showed that we could not be 95% confident that the clinic was cost-effective. Compared to the conventional group, clinic patients were three times more likely to prefer OPAC care (relative risk (RR) 2.75, 95% CI, 2.13 to 3.55; *P* <0.001) and pay more than the median WTP (US$13) for a clinic consultation (RR 3.27, 95% CI, 2.32 to 4.64; *P* <0.001).

**Conclusions:**

There is uncertainty about the cost-effectiveness of an OPAC in the Hong Kong setting. Most clinic patients were willing to pay a small amount for an anesthesia clinic consultation.

## Background

Healthcare systems of today place much emphasis on patient-centered quality outcomes and cost effectiveness. Compared to a conventional system of admitting patients at least a day before surgery, Perioperative Systems with outpatient anesthesia consultation clinics are well established in North America [1-3], Europe [4] and Australia [5,6]. While there are significant variations in the development of these Perioperative Systems between hospitals and health systems both within individual countries and between countries, this model of care involves a multidisciplinary team that provides integrated patient-focused evidence-based care from the time a decision is made that a patient should have an operation until the patient has recovered to their stable preoperative health status [7].

The benefits of establishing an outpatient anesthesia clinic (OPAC) include increasing hospital efficiency by a rapid shift from inpatient to same day admission surgery, reduction in length of hospital stay, fewer cancellations of surgery and fewer preoperative investigations [1,2,4]. While it is intuitive that re-engineering of the surgical care system should result in a substantial reduction of healthcare costs from the benefits described, there is a paucity of economic evaluations [7]. Two previous studies [8,9] suggest that the greatest gain in cost savings in the Perioperative System come from costs associated with shorter length of stay rather than from fewer preoperative investigations, but neither were formal cost-effectiveness studies.

**Figure 1:**
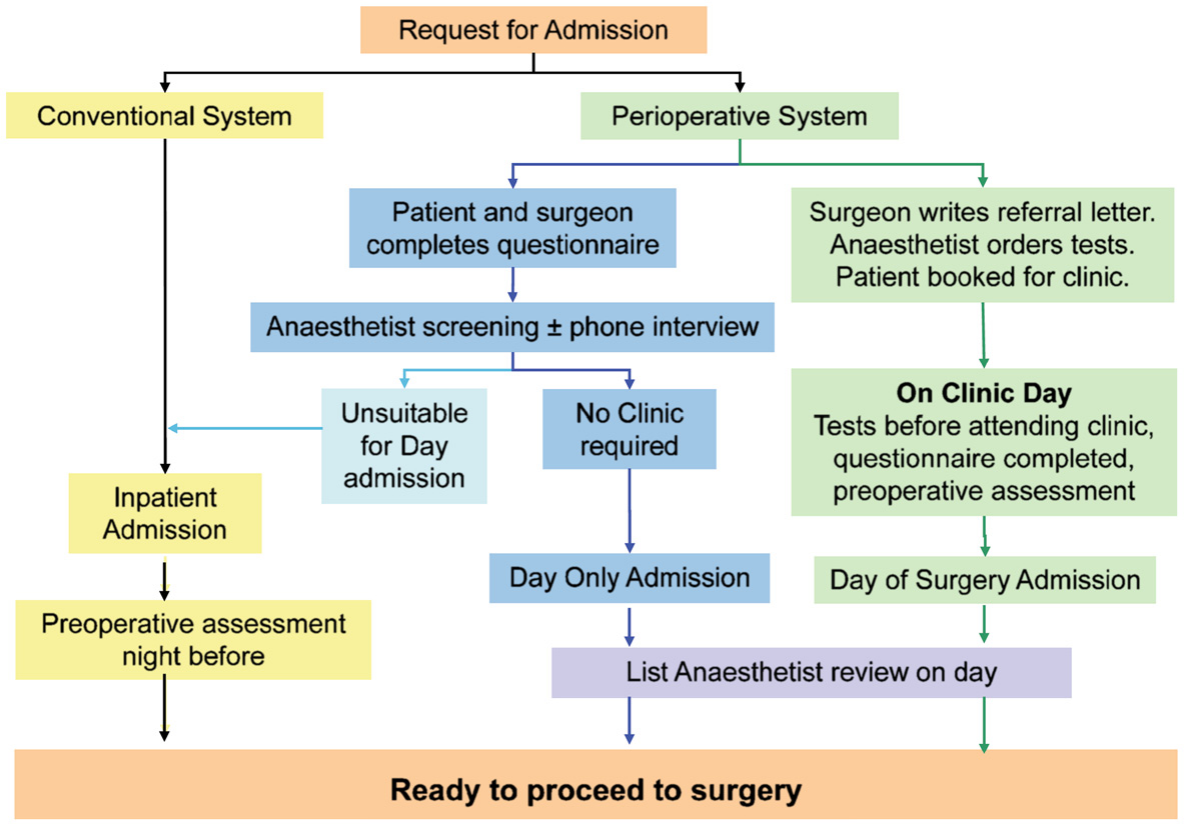
**Conventional and Perioperative System preprocedural processes at Prince of Wales Hospital, Hong Kong.** There are three types of patient groups: Day only admissions (patients admitted and discharged on the same day after elective surgery), Day of surgery admissions (patients admitted on the day of elective surgery and then stay in hospital for at least one night) and Inpatients (patients admitted before the day of surgery and then discharged on the same day or afterwards following surgery). The Perioperative System was operational in 2006 but a dedicated pre-anesthetic clinic space was not available until July 2008.

Despite the apparent benefits associated with a Perioperative System, most Hong Kong patients are admitted to public hospitals a day before surgery and then visited by an anesthesiologist for preoperative consultation on the ward. Among the few hospitals in Hong Kong with a co-existing conventional surgical system and an OPAC in place (Figure 1), the Prince of Wales Hospital established an OPAC in January 2006. From 1 January to 31 December 2009, the percentage of elective operations performed as an outpatient surgery admission and same day admission surgery were 7% and 16%, respectively (unpublished observations).

As data were required to justify any expansion of the OPAC, we performed a cost-effectiveness study on patients undergoing selective surgical procedures for which patients could be seen at either an OPAC (Perioperative System) or through the conventional system, whichever was acceptable to the surgeons. A randomized controlled trial was not possible because many surgeons were unwilling to change their admission practices and there were established clinical pathways for preoperative care for many surgical procedures in place at the time of developing the study proposal. The main objective of the matched cohort study was to compare the costs and effects of the OPAC care with the conventional approach from the perspective of the Hospital Authority (a government body funding public health services in Hong Kong). The secondary objective was to determine the patient’s Willingness To Pay (WTP) value for an OPAC.

## Methods

The study was conducted at the Prince of Wales Hospital in Hong Kong, a large university hospital. The study was approved by the local Clinical Research Ethics Committee. After written informed consent, adult patients were enrolled from 20 March 2007 to 25 November 2009. The anesthesiologist-led OPAC began operation in January 2006 but did not have its own designated office space until July 2008. It is staffed by 0.5 anesthesiologist full-time equivalent and one nurse-full time equivalent to provide the service five afternoons a week, serving an average of 8 to 10 patients per day (unpublished observations for 2010). Patients were seen in the OPAC up to three months before their surgery.

### Patients

We prospectively identified patients who underwent the following elective surgical procedures: orthopedic (total knee replacement, knee arthroscopy, anterior cruciate ligament reconstruction, total hip replacement, arthroscopic shoulder repair), gynecological (hysterectomy, salpingo-oophorectomy, cone biopsy), general (laparoscopic cholecystectomy, ligation and stripping of varicose vein, inguinal hernia repair), urological (transurethral resection of the prostate) and other (tonsillectomy, functional endoscopic sinus surgery). For these procedures, the surgeons had the option of referring their patients to the OPAC (clinic group) or through the conventional system of admitting the patient into the hospital one day before surgery (conventional group). Each clinic patient was matched by the same surgical procedure to a conventional patient on a 1:1 ratio. Selection bias could influence the association between OPAC and outcomes because high risk patients were more likely to be referred to the clinic for ‘work up’ [10]. In the design of this study, we addressed this issue by matching patients on their type of surgical procedure and adjusting for American Society of Anesthesiologists (ASA) Physical Status in statistical analyses when necessary.

Patients were expected to receive general anesthesia with or without regional anesthesia supplement. All patients received the usual care from surgeons and nursing staff in the surgical ward. Patients were excluded if they were younger than 18 years of age, undergoing emergency or obstetric surgery, or were unable to give consent. To ensure that the type of anesthesia given to patients was comparable between groups, those receiving regional or local anesthesia were not matched and were excluded from the study after reviewing their anesthetic records. Patients were also excluded from the study if they had their elective surgery cancelled before being matched to a suitable case. Data were not collected in patients who had another surgical procedure within the study period.

### Data collection

Patients in both groups were interviewed by an investigator (CHC) with a standardized questionnaire before surgery in the OPAC (clinic group) or on the ward (conventional group). We collected data on patient’s demographics, ASA physical status, cancellation of surgery on the day of intended surgery and length of hospital stay. The level of surgical invasiveness was classified as ‘minor’ (for example, hysteroscopy), ‘intermediate’ (for example, inguinal hernia repair), ‘major’ (for example, cholecystectomy) and ‘ultramajor’ (for example, total knee replacement) using the Hong Kong Government Gazette [11].

We measured the anxiety levels by asking patients to mark the visual analogue scale (VAS) on a 100 mm horizontal line with ‘not anxious at all’ at the left and ‘extremely anxious’ at the right; one for their level of anxiety about their surgical procedure and another for their level of anxiety about anesthesia. The anxiety scores were the distance in millimeters from the left side of the scales to the marks. The use of VAS to measure preoperative anxiety has been shown to be valid [12].

To measure a patient’s satisfaction with the anesthetic consultation at the OPAC or on the ward, a validated and reliable questionnaire was used [13]. The questionnaire contained five specific questions about various aspects of the anesthesia consultation and one global question on the patient’s satisfaction using a 6-point Likert scale (1 = strongly disagree to 6 = strongly agree) [13]. The total patient satisfaction score ranged from 6 to 30 and was converted to a score out of 100 for the purposes of this study. Patient satisfaction was collected immediately after the anesthesia consultation at the OPAC or on the ward. On the first day after surgery, a global measure of patient satisfaction with anesthesia care was measured by asking patients to ‘Circle the one number that describes how satisfied you are with the overall anesthetic care (before and after surgery) provided to you’ using a 5-point Likert scale (1 = insufficient, 2 = fair, 3 = appropriate, 4 = very good and 5 = excellent) [14].

### Outcomes

The primary outcome measure was the nine-item Quality of Recovery (QoR) score [15] used to measure the patient’s health-related quality of life after anesthesia on the first day after surgery. The QoR score ranged from 0 to 18 and is a valid and reliable patient-centered outcome.

Willingness To Pay (WTP) was examined to establish how patients value the different approaches to anesthetic consultation before surgery. We asked patients about their preference for the location of the anesthesia consultation (Table 1) and the maximum amount of money they were willing to pay using an open ended question format [16] for their preferred care after their consultation with an anesthesiologist. Both groups were presented with a comparison of the major differences between the two forms of anesthesia consultation during the face-to-face interview using a standardized WTP questionnaire before surgery. The likelihood of having the same anesthesiologist at both the anesthesia consultation visit and at induction, and the risk of cancellation of the surgery was based upon our empirical unpublished data. The risk of wound infection was estimated from a published study [6]. Patients were aware that HK$100 was charged for attendance at all outpatient specialist clinics in Hong Kong public hospitals. They were also aware that they were charged for admission and hospital stay (HK$50 and HK$100/day, respectively) in the public hospital system.

**Table 1:** Preference for clinic and conventional pre-anesthetic consultation

**Outpatient pre-anesthetic clinic**	**Standard inpatient surgical ward (conventional)**
See anesthesiologist up to one month before surgery as an outpatient. Less chance (10 in 100, 10%) that the clinic anesthesiologist will be the same anesthesiologist who will give you the anesthesia on the day of surgery.	See anesthesiologist one day before surgery as an inpatient. More chance (90 in 100, 90%) that this anesthesiologist will be the same anesthesiologist who will give you the anesthesia on the day of surgery.
Two visits to hospital (clinic and for surgery).	One visit to hospital for surgery.
Admission to hospital on the day of surgery.	Admission to hospital one day before surgery
Less chance (5 in 100, 5%) of surgical wound infections because overall hospital stay is shorter.	Higher chance (16 in 100, 16%) of surgical wound infections because overall hospital stay is longer.
Less chance (1 in 100, 1%) of cancellation of surgery on the day of surgery due to medical reasons.	Higher chance (5 in 100, 5%) of cancellation of surgery on the day of surgery due to medical reasons.

There are two locations in the Prince of Wales Hospital where patients can see an anesthesiologist before their surgery. One is the Outpatient Preanesthetic Clinic and the other is an Inpatient Surgical Ward. The discussion about the risks of complications and processes of anesthesia is the same at each location. The descriptions above concentrate only on the differences between these two locations.

### Costs

To perform the cost-analysis from the hospital’s perspective, the following direct costs related to anesthesia consultation were identified and summated to estimate the total direct perioperative treatment cost. First, preoperative drugs prescribed for optimizing the patient’s condition before anesthesia were extracted from the drug chart in the patient’s medical records. The unit cost was obtained from the hospital’s pharmacy. Second, the laboratory investigations ordered by the anesthesiologist before surgery during the consultation were noted in the patient’s medical records. The cost of a laboratory investigation was estimated by the unit cost from the hospital’s pathology and radiology departments. Third, the OPAC cost (HK$840) was obtained from the Hospital Authority 2008/2009 annual report [17]. Finally, we assumed the ward costs for the clinic and conventional groups on the surgical ward were the same for each day as all patients received the same care on the designated surgical wards before and after surgery. Thus, the ward cost for each patient was estimated by calculating the average hospital bed cost (HK$3,650) from the Hospital Authority 2008/2009 annual report [17]. At the time of reporting the study results, 1US$ = HK$7.78. Cancellation costs and length of hospital stay, related to a previous admission to hospital for the intended surgical procedure, were also included in the present analysis.

### Statistical analysis

We calculated that a sample size of 280 patients per group would provide 80% power to detect a difference in cost and QoR of US$128 and 0.3, respectively; the expected standard deviations of cost and QoR of US$643 and 1.2, respectively; an expected correlation of the differences of 0.1 and the maximum WTP of US$1,285 at two-tailed alpha level of 0.05 [18]. However, due to slow recruitment because of unexpected cancellation of elective surgery (62 working days) and lack of financial support for many surgical procedures to be admitted through the Perioperative System as planned during the study period, we recruited 176 patients per group.

Values are reported as mean and standard deviation (SD) or median and interquartile range (IQR). The mean difference and 95% confidence interval (95% CI) was defined as the OPAC variable of interest minus conventional variable of interest. We used matched paired *t*-test, Wilcoxon signed ranked test and McNemar’s test to compare preoperative patient characteristics between the matched groups. A multivariate analysis of variance was used to examine differences among the five components of the patient satisfaction with anesthesia consultation questionnaire [13] with a Bonferroni correction for multiple pair-wise comparisons.

We assumed that the OPAC was cost-effective if there was a reduction in the overall perioperative treatment cost per gain in QoR. Using similar methodology to our previous paper on the cost-effectiveness of an Acute Pain Service [19], a cost-effectiveness acceptability curve (CEAC) was constructed from a net benefit regression [20] to examine the probability of cost-effectiveness of an OPAC over a conventional anesthesia consultation on the ward. The CEAC is a graphical transformation from a cost-effectiveness plane, where the joint density of incremental costs and effects may straddle the northwest, northeast, southwest and southeast quadrants of the plane [21]. The construction of the CEAC was performed using the macro ‘iprogs’ available from the University of Pennsylvania (http://www.uphs.upenn.edu/dgimhsr/stat-cicer.htm; accessed May 25, 2010). A *post-hoc* sensitivity CEAC was used to determine if the OPAC was cost-effective if only preoperative costs were considered.

A Poisson regression model [22] was used to examine the factors associated with patients willing to pay more than the median WTP value for an OPAC after adjusting for the patient’s level of income, age, gender, ASA physical status and matching. We considered a two-sided *P* <0.05 to be statistically significant. All analyses were performed using STATA software version 10.1 (StataCorp, College Station, TX, USA).

## Results

Of the 676 patients screened for the study, 352 patients were matched on the type of surgical procedure in the final analysis. The remaining patients were all seen at the OPAC but not in the ward (for example, inguinal hernia repair, joint replacement, hysterectomy), or all in the ward but not at the OPAC (for example, coronary artery bypass graft surgery) during the latter half of the study.

The preoperative characteristics of the 176 matched pairs were similar (Table 2). The number of matched patients undergoing orthopaedic, general, gynaecology and urology/other procedures were 133 (76%), 24 (14%), 11 (6%) and 8 (4%), respectively.

**Table 2:** Patient characteristics

**Characteristics**	**Clinic group**	**Conventional group**	***P-*** **value**
	(n = 176)	(n = 176)	
Age, median (IQR), years	44 (28 to 59)	45 (26 to 59)	0.87
Women, number (%)	63 (35.8)	67 (38.1)	0.68
Education level, number (%)			0.20
No formal education	16 (9.1)	10 (5.7)	
Primary	36 (20.5)	42 (23.9)	
Secondary	74 (42.0)	67 (38.1)	
College	18 (10.2)	14 (7.9)	
University	32 (18.2)	43 (24.4)	
Work status, number (%)			0.54
Student	16	20	
Retired	35	44	
Employed	87	75	
Self-employed	9	9	
Unemployed	7	11	
Housewife	22	17	
Income level (US$ per month)			0.06
<$1,285	103	123	
$1,286 to $3,856	65	44	
>$3,857	7	8	
Magnitude of surgery, number. (%)			0.29
Minor	8 (4.5)	12 (6.8)	
Intermediate	26 (14.8)	21 (11.9)	
Major	48 (27.3)	44 (25.0)	
Ultramajor	94 (53.4)	99 (56.3)	
ASA physical status grade, number (%)			0.15
I	114 (64.8)	105 (59.7)	
II	56 (31.8)	60 (34.0)	
III/IV	6 (3.4)	11 (6.3)	
Duration of anesthesia, mean (SD), minutes	114 (45)	115 (44)	0.88

Patients in the clinic group had similar rates of surgery being cancelled on the scheduled date compared to the conventional group (2.3% versus 3.4% respectively, *P* = 0.75). Of the 10 cancellations of surgery, 9 (4 in clinic group, 5 in conventional group) were due to no available operating room time from the overrun of previous surgical procedures. Surgery was cancelled due to a respiratory infection in a patient belonging to the conventional group.

There was no difference between clinic and conventional groups for median levels of anxiety for surgery (26 versus 25 respectively, *P* = 0.12) or for anesthesia (20 versus 19 respectively, *P* = 0.60). Patients in the OPAC group were more satisfied with the consultation taking place without time pressure and felt more informed about their procedure than conventional patients (Table 3). When the individual components of the patient satisfaction score were summated in Table 3, the score was higher in the OPAC group (mean difference 2.10%, 95% CI: 0.51% to 3.70%). After surgery, the mean patient satisfaction with perioperative anesthesia care score out of 5 was similar between the OPAC (3.88 ± 0.73) and conventional (3.89 ± 0.74) groups (*P* = 0.94).

**Table 3:** Comparison of patient satisfaction with anesthesia consultation

	**Clinic group**	**Conventional group**	***P-*** **value**
	(n = 176)	(n = 176)	
a. Consultation took place without time pressure (mean, SD)	4.94 ± 0.69	4.76 ± 0.84	0.03
b. Explanations were easily understood (mean, SD)	5.01 ± 0.48	4.99 ± 0.53	0.67
c. Questions were clarified (mean, SD)	4.95 ± 0.70	4.89 ± 0.75	0.42
d. More informed about procedure (mean, SD)	5.05 ± 0.56	4.80 ± 0.83	<0.01
e. Process of consultation was clear (mean, SD)	4.70 ± 0.97	4.58 ± 1.01	0.26
Global satisfaction score	5.05 ± 0.44	5.00 ± 0.47	0.27
**Summated score (a to e) (%)**	82.16 ± 6.88	80.06 ± 8.27	0.01

The median length of stay in the hospital was shorter in the OPAC group than in the conventional group (three versus five days, *P* <0.001). This was due to shorter median duration of stay before surgery in the OPAC group (1, IQR zero to one day) than in the conventional group (1, IQR one to three days) (*P* <0.001). There was no difference in the median duration of stay after surgery between OPAC and conventional groups (three versus two and a half days, *P* = 0.67).

### Cost-effectiveness

The mean QoR score on the first day after surgery was similar between OPAC (13.17 ± 2.73) and conventional (13.31 ± 2.65) groups (*P* = 0.57). Table 4 shows the mean perioperative treatment costs (in $US) per patient. Of the 176 patients in the OPAC group, 81 (46%) were admitted on the day of surgery. Although the OPAC group had a significantly lower total preoperative cost than the conventional group (mean difference -$463, 95% CI: -$648 to -$278 per patient, *P* <0.01), the mean difference in the total perioperative treatment cost was not significant (−$172, 95% CI: -$684 to 340 per patient; *P* = 0.51) even after adjusting for cancellation on the day of surgery costs.

**Table 4:** Mean preoperative and overall perioperative costs ($US) per patient

**Cost category**	***Clinic group***	***Conventional group***	**Mean difference**^**a**^	***P-*** **value**
	(n = 176)	(n = 176)	(95% CI)	
a. Outpatient clinic	109.20	0	109.20	<0.001
b. Medication prescribed by anesthesiologist	0.48	0.15	0.33 (−0.18 to 0.85)	0.20
c. Investigations ordered by anesthesiologist	36.14	24.74	11.40 (0.59 to 22.19)	0.04
d. Cancellation of surgery	15.99	58.64	−42.65 (−126.49 to 41.13)	0.32
e. Inpatient bed before surgery^b^	335.87	876.99	−541.12 (−656.58 to −425.67)	<0.001
*Total preoperative cost (a to d)*	*497.68*	*960.52*	*−462.84 (−648.12 to −277.58)*	*<0.001*
f. Inpatient bed on day of surgery and afterwards	2,247.13	1,956.58	290.55 (−187.01 to 768.12)	0.23
*Total perioperative cost (a to f)*	*2,744.81*	*2,917.10*	*−172.29 (−684.48 to 339.89)*	*0.51*

^a^Negative value implies that outpatient anesthesia consultation clinic is less expensive than conventional approach; positive value implies that outpatient anesthesia consultation clinic is more expensive than conventional approach. ^b^95 patients in the clinic group were admitted at least one day before surgery into hospital.

**Figure 2:**
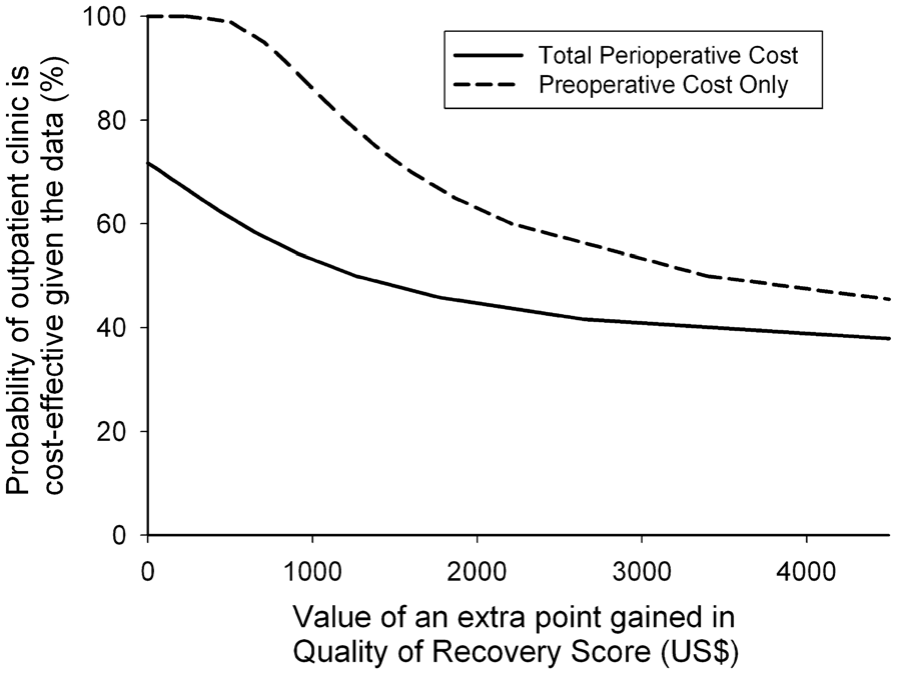
**Cost-effectiveness acceptability curves.** These were derived from comparing incremental total perioperative cost and incremental preoperative cost per incremental Quality of Recovery Score.

As there was no significant gain in QoR or significant reduction in perioperative cost in the OPAC group over the conventional group, we cannot be 95% confident that the OPAC is cost-effective. The CEAC for the observed data (Figure 2) represents the joint density (incremental cost and incremental effect) covering all four quadrants of the cost-effectiveness plane, with the curve suggesting that most densities fell within the southwest quadrant (less costly, less effective). As the CEAC did not cut the y-axis at 0, some of the density involved cost-savings (71% in the southeast or southwest quadrants). As the CEAC for the observed data did not asymptote to 100, only 42% of the density involved QoR gains.

When only preoperative costs were considered, the CEAC showed that the joint density fell within the southeast and southwest quadrants (less costly, more or less effective; that is, 100% cost-savings). The CEAC for preoperative cost also shows that we can be confident that the conventional system is not cost-effective compared to the OPAC when the decision maker is willing to pay less than US$596 for an extra unit gain in QoR per patient; if the decision maker is willing to pay above US$596 for an extra unit gain in QoR per patient, then we cannot be 95% confident that the two systems differ in value.

### Willingness to pay

As expected, patients in the OPAC group preferred to have their consultation at the clinic (75%) than on the ward (11%), with 14% indicating no preference for either location. More patients in the conventional group preferred to have their consultation on the ward (37%) than at the clinic (27%), with 36% indicating no preference for either location. Thus, clinic patients were three times more likely to prefer OPAC care (relative risk (RR) 2.75, 95% CI: 2.13 to 3.55; *P* <0.001) than conventional patients. The median (IQR) WTP for an OPAC and conventional ward anesthesia consultation were US$12.85 ($7.71 to $12.85) and US$12.85 ($0 to $25.71) respectively, regardless of which group the patient belonged to. However, compared to conventional patients, OPAC patients were more likely to pay more than US$12.85 for a clinic consultation (RR 3.27, 95% CI: 2.32 to 4.64; *P* <0.001) after adjusting for income, gender, age and ASA Physical Status score.

## Discussion

We conducted a formal cost-effectiveness study using matched patients undergoing elective surgery through the conventional and Perioperative Systems running concurrently. Previous cost-analysis studies [8,9] have quantified the cost savings associated with establishing a Perioperative System but were likely to be biased because of the before-after study design used.

Although the length of stay was shorter in the OPAC group, this did not translate to significant cost savings in favor of the Perioperative System. Even after the patients had their anesthesia consultation at the OPAC, surgeons continued to admit more than half the group into hospital at least one day before surgery to occupy a hospital bed and a place on the operating list. In many developed Perioperative Systems, this practice would be obsolete unless the patient was at very high risk of postoperative complications.

It should be noted that postoperative bed cost accounts for approximately 75% of the total perioperative cost. This explains why we failed to find the OPAC to be cost-effective when all direct costs before and after surgery were summated. However, if all patients in the OPAC group were admitted on the day of surgery, the total perioperative cost-savings of US$525 (95% CI: $18 to $1,033) per patient would be significantly in favor of the Perioperative System (*P* = 0.04). Compared to the conventional system, this would translate to an 18% reduction in overall perioperative treatment costs, a similar finding to Boothe and Finegan’s study (18% reduction) [8]. The implication of this analysis suggests that there are some barriers to adopting the OPAC approach in some of our surgeons. These may include their traditional belief that OPAC patients are ‘not ready’ on the day of surgery and their prevailing practice of admitting an OPAC patient overnight before surgery [23]. Continued efforts are ongoing by anesthesiologists and surgeons to promote appropriate referral of patients to the OPAC and timely hospital admission on the day of surgery of OPAC patients. We believe that a 10% to 20% reduction in the total perioperative cost per patient associated with our Perioperative System is highly feasible in this setting in line with overseas experience [7].

Caution is needed in the interpretation of our sensitivity analysis (Figure 2) that included only the preoperative cost as it made up a small proportion (approximately 25%) of the overall perioperative cost. The implication of these results highlights the need for financial incentives to be put in place to encourage shorter length of stay, especially after surgery. This may include the Pay for Performance diagnosis-related case-mix model that has recently been introduced into the Hong Kong public hospital funding allocation scheme [24].

The surgical system at the Prince of Wales Hospital in Hong Kong is unique with a hybrid model of the new and old systems of surgical care (Figure 1). This has been due in part to a number of factors. First, the incentive to maximize the bed occupancy in Hong Kong public hospitals is less than in similar Australian hospitals as the number of acute hospital beds per population is higher (2.9 [25] versus 2.6 [26] per 1,000, respectively). Also the lack of a centralized preoperative holding area has impeded the use of ‘hot bedding’ whereby patients do not require a ward bed until the latter part of the morning, enabling the bed to be fully utilized overnight. Second, the role of the community primary healthcare team in Hong Kong [27] for supporting the patient before and after surgery is less defined than in the Australian setting. Despite favorable patient attitudes towards day case surgery in Hong Kong [28], most elective surgery cases are booked and performed under individual surgical specialty teams who look after their own operating room lists rather than through a centrally organized and integrated Perioperative Service. These unique factors produce particular challenges to implementing the complete Perioperative model in Hong Kong and potentially limit the stakeholders from deriving the benefits associated with such a system.

From the patient’s perspective, both groups valued the two approaches to anesthesia consultation equally at an average WTP value of US$13. Despite careful wording of the WTP question, the low WTP value may reflect the actual charge that patients currently pay for specialist-based outpatient clinics in Hong Kong and a high patient expectation of the government to heavily subsidize public health services. Nevertheless, those who were seen at the OPAC were three times more likely to both prefer it and pay more than US$13 for this type of care over the conventional approach. Irrespective of where anesthesia consultation takes place, patients in a German study were found to value a close relationship with an anesthesiologist without waiting too long to see them [29]. Of the €100 available to spend, patients were willing to pay €36 for a pre-anesthetic visit performed by the anesthesiologist who would give anesthesia, €26 to wait less than two hours, €16 for preferred location of pre-anaesthetic visit, €12 for multimedia information and €10 for ambience [29].

The strong preference for an OPAC may be associated with a marginally higher preoperative patient satisfaction level in clinic patients than those in the conventional system. OPAC patients were more informed about the risks and processes of anesthesia without constraints on the length of their consultation. Our findings are consistent with high levels of patient satisfaction with the information from, and sufficient time with, an anesthesiologist at an OPAC [13,30]. As in a previous study [14], we found no difference in the overall perioperative patient satisfaction ratings for anesthesia care between groups.

Previous studies [1,4,31,32] have consistently shown a significant decrease in the rate of cancellation of surgery associated with a Perioperative System. However, as our rate of surgery cancellations was already low in OPAC patients, we did not find a significant difference between groups. The majority of these cancellations were related to operating room lists overrun rather than inadequately prepared patients, a similar finding to a recent study [33]. Many surgeons scheduled their OPAC patients in the later part of the operation list to allow more time for pre-procedural processes to occur but often underestimated the length and/or complexity of the procedures of other patients at the start of the operating list. Strategies to reduce the rate of cancellations from cases running over the allocated time may improve the overall operating room efficiency and reduce healthcare costs.

There are several limitations to this study. In calculating the direct costs of both approaches to anesthesia consultation, the net costs related to better prepared patients and perioperative complications costs were not accounted for. Previous studies have shown that patients in the Perioperative System are better prepared for surgery [10] and may have less risk of wound infections [6], implying that our CEAC may be imprecise. Despite the lack of randomization in this study, the ASA physical status was similar between matched patients; indirect evidence (median postoperative length of hospital stay) suggests that OPAC patients were at no greater risk of complications after surgery than conventional patients.

The results of this study may not be generalizable to different organizational structures of OPAC outside Hong Kong. However, it highlights the difficulties in changing hospital organization culture, clinical practice and behaviors when implementing a new Perioperative System. In many centers elsewhere, a nurse-led OPAC can provide a similar service without affecting patient satisfaction levels [30] and patient safety [34] but we are unaware of any published formal cost-effectiveness analysis using this model.

## Conclusions

In conclusion, the cost-effectiveness of the OPAC in Hong Kong remains uncertain because significant reductions in the preoperative costs make up a small proportion of the overall perioperative treatment cost. Nevertheless, OPAC patients had a strong preference for where the anesthesia consultation should take place, were willing to pay a small amount for it, and were more informed about the risks and processes of anesthesia. Encouraging more surgeons to use our OPAC may help address the expected growth in the number and complexity of surgical procedures being performed on older and sicker patients, with increasing awareness of and demand for high quality and safe care, in a setting of limited healthcare resources.

## Abbreviations

ASA = American Society of Anesthesiologists; CEAC = cost-effectiveness acceptability curve; OPAC = outpatient anaesthesia clinic; QoR = quality of recovery; VAS = visual analogue scale; WTP = willingness to pay.

## Competing interests

The authors declare that they have no competing interests.

## Authors’ contributions

AL conceived of the study, designed the study, analyzed and interpreted the data, and drafted the manuscript. PTC, TG and AMHH participated in the study design and interpretation of the data. CHC collected the data. PTC, CHC, TG and AMHH helped revise the manuscript. All authors read and approved the final manuscript.
